# Comparison of Recurrence and Quality of Life Between Myoma Embolization and Myomectomy

**DOI:** 10.7759/cureus.40372

**Published:** 2023-06-13

**Authors:** Hilal Aktürk, Mustafa Cengiz Dura, Berk Gürsoy, Faruk Ikizoğlu, Erkan Göl, Waseem O.I. Alsalamin, Murat Ekin

**Affiliations:** 1 Obstetrics and Gynaecology, Bakirkoy Dr. Sadi Konuk Training and Research Hospital, Istanbul, TUR; 2 Medicine, University of Health Sciences, Istanbul, TUR; 3 Medicine, Al-Quds University, Abu Dis, PSE

**Keywords:** recurrence, myomectomy, uterine myomas, quality of life, embolization

## Abstract

Introduction: Uterine myomas represent the most frequently diagnosed tumors among women of childbearing age. Symptoms often include profuse menstrual bleeding, diminished quality of life, and in some cases, infertility. The size and position of the fibroids typically influence the condition's manifestations. Moreover, symptomatology often varies depending on the fibroids' location. This investigation aimed to discern if there exists a significant correlation between life quality, reoccurrence rate, quality of life, and recurrence levels among patients who have undergone myomectomy and uterine fibroid embolization, respectively.

Methodology: A retrospective cross-sectional study was conducted to compare the rates of recurrence and impacts on life quality between uterine fibroid embolization and myomectomy in women diagnosed with uterine myomas. Data were collected from 152 women who sought treatment at the Obstetrics and Gynecology clinic and also the Interventional Radiology clinic between January 2009 and January 2021. Thirteen participants were excluded due to the inability to maintain contact. The trial encompassed 76 patients who underwent myomectomy and 63 who had uterine fibroid embolization. In both groups, the life quality of 50 patients, five years postsurgery, was assessed using the UFS-QOL measure. Eligible participants were females between 20 and 40 years, with symptomatic Type 3-5 fibroids as per the FIGO classification, and with no comorbidities. Individuals under 20 or over 40 years, or those with fibroids classified as FIGO types 1,2,6,7,8, were not included. Other exclusion criteria included pregnancy status, abnormal endometrial biopsy results, abnormal smear tests, polyps, cancer, adenomyosis and coagulation disorders.

Results: The recurrence of fibroids was identified through symptomatology and diagnostic radiological methods. The recurrence rate was found to be 31.6% (n=24) for myomectomy patients and 14.3% (n=9) for those who underwent uterine fibroid embolization, with no statistically significant difference between the two groups (p > 0.05). The group subjected to myomectomy exhibited fewer symptoms, lower anxiety, and better physical mood scores. The myomectomy group displayed higher average anxiety scores (p<0.01). There were no significant disparities in control, consciousness, sexual function, or overall scores between the two groups. Symptoms and anxiety saw a marked reduction in the first postoperative year compared to the preoperative period (p<0.01). Compared to presurgery, energy, mood, awareness, and sexual function exhibited significant improvements in the first and fifth postoperative years (p<0.01).

Conclusions: Our findings suggest a nonsignificant recurrence rate in the myomectomy group compared to the uterine artery embolization group. Notably, the decrease in symptom occurrence and anxiety following myomectomy was significantly favorable in terms of quality of life. While embolization was offered as a therapeutic option, myomectomy yielded more favorable results concerning quality of life.

## Introduction

Uterine myoma is a prevalent condition affecting women primarily between the ages of 30 and 55, and it is the leading cause of surgical intervention in both developed and emerging nations [1-3]. Age remains a critical factor, with myomas escalating between 40 and 50 [4].

The location and size of the myoma, along with the clinical symptoms it induces, are pivotal in defining the therapeutic approach. The common patient-reported issues include infertility history, desire for pregnancy, compression symptoms, heavy menstrual bleeding, and pelvic pain, guiding the selection of appropriate treatment modalities [5].

Nonsurgical treatment options include GnRH analogs, oral contraceptives, hormone-based preparations, levonorgestrel-releasing intrauterine devices, uterine artery embolization, focused ultrasound surgery, endometrial ablation, and radiofrequency fibroid ablation. For surgical treatment, conventional methods and laparoscopic and robot-assisted procedures can be considered [6].

Academic literature explores the quality of life of patients following uterine artery embolization and myomectomy, along with the recurrence rate postprocedure. Recurrence is typically symptomatic and accompanied by the detection of a new myoma via radiological imaging; a repetition of the procedure may accompany it. Understanding patient satisfaction, the associated costs of hospitalization, and the expenses linked to these procedures is invaluable in selecting the most suitable treatment when a patient seeks hospital care. So, we wanted to use the resources of our existing institution for this purpose.

## Materials and methods

Study protocol

Information was collected through hospital data and questionnaires between 2009 and 2021 at the Sadi Konuk Training and Research Hospital in Bakırköy. This single-center retrospective study included 152 patients who sought services at the Gynecology and Interventional Radiology clinics. During the investigation, 13 patients' records could not be accessed. The research received approval from the ethics committee of Bakırköy Dr. Sadi Konuk Training and Research Hospital (Protocol code: 2022/17). The hospital database served as a source to collect the patient's demographic data, and the UFS-QOL questionnaire was used to gather information on their quality of life. The requisite permissions to use the validated scale form were obtained from Dr. Burcu Cengiz, a faculty member. We collected patient data from the hospital database, which included pre and postprocedure magnetic resonance imaging (MRI) reports, findings from transvaginal ultrasonography, and patient examination records.

Inclusion criteria were defined as age between 20 and 40 years and symptomatic Type 3-5 uterine myomas per FIGO classification. Exclusion criteria were as follows: FIGO classified Type 1-2-6-7-8 myomas, polyps, adenomyosis, neoplasia, coagulation abnormalities, abnormal Pap smear, and abnormal endometrial biopsy findings, and age less than 20 or greater than 50. To rule out the diagnosis of adenomyosis, consultation with radiology specialists was made using ultrasound and MRI diagnostic methods.

The UFS-QOL questionnaire was employed to evaluate the participants. This assessment was administered presurgery, one-year postsurgery, and again five years later. Initially, ultrasonography imaging is preferable-frequent urination following constipation. In cases of sexual pain or distress, MRI position verification is preferred. Patients were offered both options. The study focused on patients who underwent myomectomy and uterine fibroid embolization.

Statistical analysis

We examined the normality assumptions of the data using the Kolmogorov-Smirnov test. The descriptive statistics for continuous variables comprised the mean, standard deviation, median, minimum, and maximum values. To compare variables between the myomectomy and uterine artery embolization groups, we employed the Mann-Whitney U test, and for within-group comparisons, we utilized the Friedman test. If a significant difference appeared in the within-group comparison, we concluded that patients were offered both options. The study focused on patients who underwent myomectomy and uterine fibroid embolization. Posthoc tests with Bonferroni correction were performed, setting the p-value at 0.05/3 = 0.17. We considered a p-value of less than 0.05 as statistically significant.

## Results

The distribution of the participants within the scope of the research is given in Tables 1, 2. When Table 2 is examined, the localization of myoma was found to be posterior (n:42, 55.3%) and anterior (n:34, 44.7%) for all participants who underwent myomectomy, respectively. According to the number of births, most (n=39, 51.3%) of the participants had one birth, then two (n=26, 34.2%) and three (n=10, 13.2%) births, while the least (n=1, 1.3%) appears to have had four births (Table 1).

**Table 1:** Demographic Data of Patients Who Underwent Myomectomy

Parameters	Mean Size	%
Number of Birth and Rate		
1	39	51.3
2	26	34.2
3	10	13.2
4	1	1.3
Surgical History		
	66	86.8
None		
C-section	10	13.2

**Table 2:** Morphological, Anatomical and Surgical Data of The Patient Who Underwent Myomectomy

Parameters	Mean Size	%
Myoma Localization	76	100
Posterior	42	55.3
Anterior	34	44.7
Operation History		
Laparoscopic Myomectomy	35	46.1
L/S Myoma and Endometriosis Surgery	18	23.7
Robotic Assisted Myomectomy	7	9.2
L/S Myoma and Cystectomy	4	5.3
Laparotomic Myomectomy	12	15.8
Myoma Type		
Type 3	6	7.9
Type 4	40	52.6
Type 5	30	39.5
Myoma Size		
>5cm	28	36.9
5-10cm	43	56.6
>10cm	5	6.5
Number of Myoma		
1	47	61.8
2	28	36.8
3	1	1.3

According to the previous operation, 'Laparoscopic Myomectomy' was the most (n=35, 46.1%) followed by 'Laparoscopic Myomectomy and Endometrioma Surgery' (n=18, 23.7%) and at least (n=4, 5.3%) ' Laparoscopic Myomectomy and 'Laparotomic Myomectomy' (n=12, 15.8%) and 'Robotic Assisted Myomectomy' (n=7, 9.2%) are seen.

Participants in the study were categorized according to the FIGO Classification as Type 3 (n=40, 52.6%), Type 4 (n=30, 39.5%), and Type 5 (n=6, 7.9%). When analyzed according to myoma size, the most frequent was 2 cm (n=12, 15.8%), followed by 6 cm (n=11, 14.5%), with 4 cm, 5 cm, and 7 cm each presenting in 10 cases (13.2%). As for the number of myomas, the majority of participants had a single fibroid (n=47, 61.8%), trailed by those with two fibroids (n=28, 36.8%), while a minority presented with three fibroids (n=1, 1.3%).

A review of the surgical histories revealed that most subjects (n=66, 86.8%) had no previous surgeries, while a smaller group (n=10, 13.2%) had experienced Cesarean sections. The participants' mean age was found to be 39.21 years. The mean size of the myomas was established as 5.95 cm.

Among the participants who received fibroid embolization, the location of myomas was primarily posterior (n = 43, 68.3%) and anterior (n = 20, 31.7%), as outlined in Table 3.

**Table 3:** Demographic Data of Patients Who Underwent Angiographic Myoma Embolization

Parameters	Mean Size	%
Birth Number		
1	29	46.0
2	20	31.7
3	12	19.0
4	2	3.2
Surgical History		
None	45	71.4
C-Section	11	17.5
Laparoscopy	7	11.1

Regarding the number of births, a significant proportion of participants had given birth once (n = 29, 46%), followed by those who had given birth twice (n = 20, 31.7%) and thrice (n = 12, 19%). A small fraction had four children (n = 2, 3.2%). Every participant (n = 63, 100%) underwent angiographic myoma embolization.

Type 3 (n = 40; 63.5%), Type 4 (n = 20; 31.7%), and Type 5 (n = 3; 4.8%) myomas were found in a majority of the participants. Myoma sizes of 6 cm were observed most frequently (n = 20; 31.7%), followed by 7 cm (n = 12, 19%) and 5 cm (n = 6, 13.2%).

In terms of surgical history, most participants (n=45, 71.4%) had no prior surgeries. The next most common procedures were Cesarean sections (n=11, 17.5%) and laparoscopies (n=7, 11.1%).

Examining Tables 4, 5, it is evident that myoma recurrence occurred in the patient groups (n=24, 31.6%) who underwent myomectomy for myoma diagnosed by patient examination findings, transvaginal USG image, and MRI. Among patients who underwent angiographic myoma embolization, the rate of myoma regrowth or recurrence was 14.3% (n=9). The p-value for the analysis of the two tables was 0.132. No significant difference existed.

**Table 4:** Recurrence Rate of Procedures After Angiographic Fibroid Embolization

Parametreler	Mean Size	%
Recurrence of symptoms	9	14.3
No recurrence	54	85.7

**Table 5:** Recurrence of Symptoms After Myomectomy in Patients Who Underwent Myomectomy

Parameters	Mean Size	%
Recurrence of symptoms	24	31.6
No recurrence	52	68.4

Comparing myomectomy and myoma embolization, the average hospital stay for myomectomy was 1.83 days, whereas myoma embolization required only 0.83 days (Table 6).

**Table 6:** Difference in Hospitalization Duration Between the Two Operations

	Hospitalization (Day)	
Operation Type	Mean Value	Standart Deviation (s)
Myoma Embolization	0.83	0.34
Myomectomy	1.83	0.74

Comparing the subdimensions and total scores of the myoma uteri symptom and quality of life scale between groups

**Figure 1:**
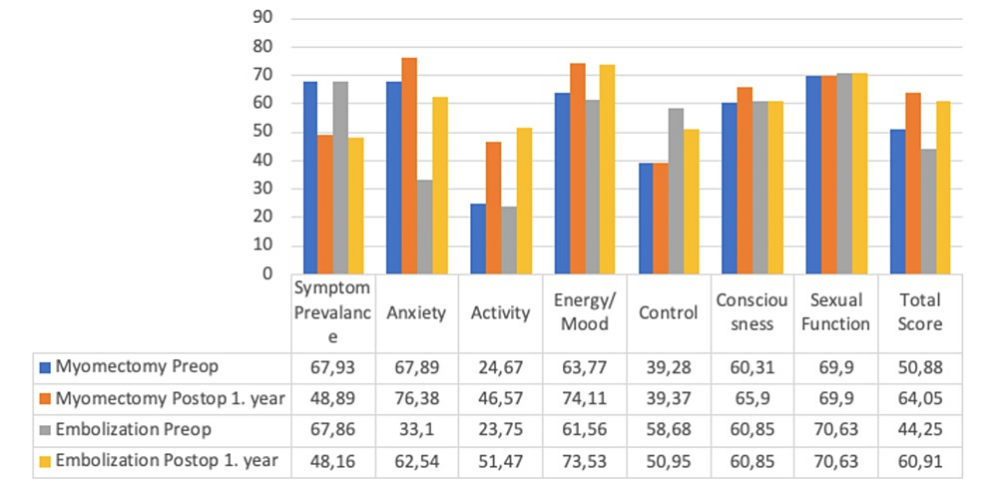
Comparative Analysis of Scoring Systems for Myomectomy and Myoma Embolization Groups

Within the myomectomy and myoma embolization scheme, both the sub-scores and overall scores of the uterine myoma symptom and quality of life surveys were evaluated both before surgery and postoperatively at the one-year and five-year marks, as demonstrated in Table 7 and Figure 1.

**Table 7:** Comparison of the Sub-Dimensions and Total Score of the Myoma Uteri Symptom and Quality of Life Scale Within and Between Groups

	Preop		Postop 1Year		Postop 5. Year		
Groups	Mean. ± SS.	Median(Min. - Max.)	Mean. ± SS.	Median (Min. - Max.)	Mean. ± SS.	Median (Min. - Max.)	With in Group Differences
Symptom Severity							
Myomectomy	67.93 ± 9.78	68.75 (46.88 - 90.63)	48.89 ± 10.44	53.13 (21.88 - 78.13)	45.25 ± 12.63	50.00 (21.88 - 62.50)	χ2=80.79, P<.001
Embolization	67.86 ± 9.66	68.75 (46.88 - 90.63)	48.16 ± 10.55	46.88 (28.13 - 75.00)	46.94 ± 10.11	46.88 (28.13 - 75.00)	χ2=88.82, P <.001
Difference between groups	U=2381.5, P= .958		U=2178.00, P=.358		U=1214.00, P=.803		
Anxiety							
Myomectomy	67.89 ± 14.61	70.00 (25.00 - 95.00)	76.38 ± 13.38	80.00 (45.00 - 100.00)	77.00 ± 10.05	80.00 (55.00 - 100.00)	χ2=2.02, P=.364
Embolization	33.10 ± 9.18	35.00 (5.00 - 55.00)	62.54 ± 8.56	65.00 (40.00 - 80.00)	81.80 ± 9.99	80.00 (60.00 - 100.00)	χ2=97.50, P<.001
Difference between groups	U=158.50, P<.001		U=948.50, P<.001		U=940.00, P=.030		
Activity							
Myomectomy	24.67 ± 10.61	25.00 (3.57 - 46.43)	46.57 ± 12.54	46.43 (21.43 - 71.43)	47.14 ± 10.77	50.00 (21.43 - 71.43)	χ2=86.43, P<.001
Embolization	23.75 ± 10.02	21.43 (7.14 - 46.43)	51.47 ± 12.87	50.00 (21.43 - 82.14)	50.07 ± 10.39	46.43 (32.14 - 71.43)	χ2=92.64, P<.001
Difference between groups	U=2254.00, P=.551		U=1896.50, P=.035		U=1170.50, P=.580		
Energy/Mood							
Myomectomy	63.77 ± 17.92	71.43 (35.71 - 85.71)	74.11 ± 13.55	78.57 (39.29 - 96.43)	76.14 ± 12.19	82.14 (42.86 - 89.29)	χ2=52.37, P<.001
Embolization	61.56 ± 18.55	71.43 (35.71 - 85.71)	73.53 ± 11.31	75.00 (50.00 - 96.43)	70.57 ± 12.30	69.64 (50.00 - 96.43)	χ2=63.093, P<.001
Difference between groups	U=2275.00, P=.611		U=2215.00, P=.446		U=914.50, P=.020		
Control							
Myomectomy	39.28 ± 25.53	45.00 (-5.00 - 75.00)	58.68 ± 19.21	65.00 (5.00 - 85.00)	65.80 ± 16.46	70.00 (15.00 - 85.00)	χ2=23.52, P<.001
Embolization	39.37 ± 23.43	40.00 (.00 - 75.00)	50.95 ± 24.24	50.00 (.00 - 85.00)	57.60 ± 20.98	65.00 (5.00 - 85.00)	χ2=62.08, P<.001
Difference between groups	U=2354.00, P=.865		U=1988.50, P=.085		U=968.50, P=.051		
Consciousness".							
Myomectomy	60.31 ± 26.24	41.67 (25.00 - 100.00)	65.90 ± 22.94	62.50 (25.00 - 100.00)	65.67 ± 22.69	58.33 (25.00 - 100.00)	χ2=9.31, P=.010
Embolization	60.85 ± 26.67	41.67 (25.00 - 100.00)	60.85 ± 26.67	41.67 (25.00 - 100.00)	61.83 ± 27.31	41.67 (25.00 - 100.00)	χ2=.00, P=1.00
Difference between groups	U=2345.00, P=.827		U=2052.50, P=.137		U=1117.00, P=.339		
Sexual Function							
Myomectomy	69.90 ± 21.33	75.00 (25.00 - 100.00)	69.90 ± 21.33	75.00 (25.00 - 100.00)	68.25 ± 22.05	50.00 (25.00 - 100.00)	χ2=.13, P=.936
Embolization	70.63 ± 19.33	75.00 (25.00 - 100.00)	70.63 ± 19.33	75.00 (25.00 - 100.00)	72.00 ± 16.08	75.00 (50.00 - 100.00)	χ2=7.200, p=.027
Difference between groups	U=2341.50, P=.819		U=2341.50, P=.819		U=1084.00, P=.237		
Total Score							
Myomectomy	50.88 ± 9.58	51.72 (30.17 - 68.97)	64.05 ± 8.98	64.22 (48.28 - 81.03)	65.88 ± 7.40	65.52 (48.28 - 79.31)	χ2=71.00, p<.001
Embolization	44.25 ± 8.81	43.10 (30.17 - 65.52)	60.91 ± 8.30	59.48 (45.69 - 77.59)	64.52 ± 6.97	64.22 (50.86 - 79.31)	χ2=92.86, p<.001
Difference between groups	U=1438.50, P<.001		U=1945.50, P=.058		U=1092.00, P=.276		

## Discussion

With the comparison of recurrence rates and quality of life between myoma embolization and myomectomy, we can start by asserting that both procedures serve as therapeutic options for uterine myoma, a common condition affecting women of reproductive age. The choice between these treatments often depends on the clinical context, including the size and location of the fibroids, the patient's symptoms, age, and desire for future fertility [7]. 

Research has shown that both treatments can be effective in reducing the size of fibroids and alleviating associated symptoms. However, they have different impacts on recurrence rates and the patient's quality of life. In our study, we have aimed to explore these differences in greater detail. 

The findings of our investigation suggest that there was no notable statistical difference between the two groups who returned to the hospital with symptoms postprocedure and had radiological evidence of fibroid recurrence. This indicates that both uterine fibroid embolization and myomectomy were comparatively successful in preventing fibroid recurrence. These outcomes align with prior studies that reported similar recurrence rates for both treatments [8]. 

When we looked up the literature, the preoperative and postoperative comparison revealed that the total quality of life score increased in both groups after the procedure [8]. However, the score was higher in the group that underwent myomectomy. The hospital stay was reported to be myoma embolization was detected shorter than myomectomy (two days versus four days), with angiographic myoma embolization having a higher recurrence rate; there was no significant difference between the two groups based on UFS-QOL scores. Patients who underwent angiographic myoma embolization had significantly higher scores for symptom severity, whereas myomectomy patients had significantly higher scores for anxiety, control, and HRQL [8- 12]. 

In our study, the mean preoperative anxiety and HRQ total score of the myomectomy group were statistically substantially higher regarding quality of life. In contrast, no statistically significant differences were found between the groups regarding symptom severity, activity, vitality, temperament, control, consciousness, sexual function, or total score. Compared to the preoperative period, symptom severity and anxiety were substantially reduced one year after surgery. Energy, temperament, consciousness, and sexual function were substantially greater in the preoperative period than in the first and fifth years. 

Preoperative, postoperative first-year, and postoperative fifth-year procedures have been evaluated independently and contribute to the literature in light of previous research. We have quantitative test results regarding the quality of life and the extent to which patients will benefit from a given procedure. 

In our study, it was determined that the recurrence rate with imaging method verification was higher than embolization in the myomectomy group after symptomatic (heavy menstrual bleeding, urinary symptoms, and pressure symptoms) admission to the hospital. During the first year and fifth year following the operation, recurrence of uterine fibroids (over 1 cm in size) was identified through routine gynecological examinations conducted with ultrasound guidance.

Our hospital stays are shorter than those described in the literature, and optimal clinical well-being and pain control are prerequisites for discharge [9-11]. This finding underscores the importance of considering not just the medical outcomes but also the patient's subjective experiences when deciding on a treatment strategy. Myomectomy, despite being a more invasive procedure, may be more suitable for certain patients due to its positive impact on quality of life. 

It is important to note that these results are based on a specific sample, and further research would be beneficial to validate these findings in larger, more diverse populations. In our hospital, patients were informed about the postembolization procedure, and discharge was planned accordingly. The embolization procedure does not have a preoperative and postoperative MRI protocol. Patients experiencing pressure symptoms and back pain are given preference for this procedure. Additionally, future studies might explore the potential reasons behind these differences in quality-of-life outcomes, such as the impact of surgical invasiveness, recovery time, and possible complications on patients' mental and physical well-being. 

## Conclusions

The research found that patients who underwent myomectomy had a recurrence rate of 31.6% (n=24) for uterine fibroids, while those who had angiographic myoma embolization had a substantially lower recurrence rate of 14.3% (n=9). These differences between the two patient groups require further investigation, with the longer hospitalization duration associated with myomectomy being one notable distinction. The study analyzed the preoperative, one-year postoperative, and five-year postoperative values of the patients. Although the average anxiety score was significantly higher in the myomectomy group compared to the control group, there were no statistically significant variations in symptom severity, activity levels, vitality, temperament, control, awareness, sexual function, or overall score between the two groups. During the first year following surgery, there was a notable decrease in symptom severity and anxiety levels compared to the preoperative period. Moreover, significant improvements were observed in energy levels, temperament, control, consciousness, and sexual function during the first and fifth years after the surgery, highlighting their importance in understanding the patient experience and surgical outcomes.
